# Structural and Thermal Characterisation of Nanofilms by Time-Resolved X-ray Scattering

**DOI:** 10.3390/nano9040501

**Published:** 2019-04-01

**Authors:** Anton Plech, Bärbel Krause, Tilo Baumbach, Margarita Zakharova, Soizic Eon, Caroline Girmen, Gernot Buth, Hartmut Bracht

**Affiliations:** 1 Institute for Photon Science and Synchrotron Radiation, Karlsruhe Institute of Technology, D-76021 Karlsruhe, Germany; baerbel.krause@kit.edu (B.K.); tilo.baumbach@kit.edu (T.B.); gernot.buth@kit.edu (G.B.); 2 Laboratory for Applications of Synchrotron Radiation, Karlsruhe Institute of Technology, D-76021 Karlsruhe, Germany; 3 Institute of Microstructure Technology, Karlsruhe Institute of Technology, D-76021 Karlsruhe, Germany; margarita.zakharova@partner.kit.edu; 4 Institute of Materials Physics, University of Muenster, D-48149 Münster, Germany; soizic.eon@laposte.net (S.E.); girmen@uni-muenster.de (C.G.); bracht@uni-muenster.de (H.B.)

**Keywords:** thin films, multilayers, thermal conductivity, thermal expansion, laser heating, synchrotron pump-probe powder scattering

## Abstract

High time resolution in scattering analysis of thin films allows for determination of thermal conductivity by transient pump-probe detection of dissipation of laser-induced heating, TDXTS. We describe an approach that analyses the picosecond-resolved lattice parameter reaction of a gold transducer layer on pulsed laser heating to determine the thermal conductivity of layered structures below the transducer. A detailed modeling of the cooling kinetics by a Laplace-domain approach allows for discerning effects of conductivity and thermal interface resistance as well as basic depth information. The thermal expansion of the clamped gold film can be calibrated to absolute temperature change and effects of plastic deformation are discriminated. The method is demonstrated on two extreme examples of phononic barriers, isotopically modulated silicon multilayers with very small acoustic impedance mismatch and silicon-molybdenum multilayers, which show a high resistivity.

## 1. Introduction

Nanoscale analysis of materials profits from the high brilliance of synchrotron-based sources by e.g., real-space or reciprocal space methods. Apart from the high resolution in real and reciprocal space the time structure of light emission at synchrotrons adds the ability to investigate dynamic processes, such as lattice motion or dissipation. One particularly important aspect of many functional materials on the nanoscale are thermal properties. In a large class of applications, such as in semiconductor integrated circuits, it is important to dissipate heat efficiently to limit heat-induced damages. On the other hand, heat conduction is an unwanted dissipation process for thermoelectric devices, which reduces the efficiency of charge collection through thermopower [1,2]. In the second case, optimization of thermoelectric materials involved minimization of the phonon contribution to thermal transport [3,4,5]. This can happen through introduction of interfaces with acoustic impedance mismatch or defects (pointlike or particles) [6,7,8], which increase phonon scattering. Multilayers, in particular, represent an interesting class of phonon barriers. The periodicity of stacking perpendicular to the surface leads to a modification of phonon propagation that can be interpreted as a new Brillouin zone, leading to phonon reflection and band bending [9,10,11]. Consequently, the pass band for phonons may be modified further than just by introducing individual interfaces. In analogy to optical coating a stop band may be forming [12,13,14], but for short periods Bloch-wave like phonon transmission can set in [15,16]. The case of layered isotopic substitution drew attention due to the fact that such crystals may electronically behave as bulk, but modulate phonon transport [14,17].

Consequently, a precise evaluation of thermal conduction on a nanoscale is very important. On surfaces several approaches are being used, the 3ω method that explores the dependence of the electrical conductivity of conducting structures during alternating-current resistive heating on top of the investigated structures as well as the dependence of optical reflectivity of transducer layers on temperature (TDTR) [18,19,20]. Both analyze the cooling time scale of the thermal conductivity in the structure below the surface. In a similar way, cooling of nanostructures can be sensed after pulsed heating to evaluate thermal transport in bulk-like conditions [21,22,23]. While optical methods are readily available in laboratories, we describe the same concept of pulsed laser heating, but probing the temperature by X-ray scattering, which adds better access to nanoscale structure [24,25,26,27]. X-ray scattering can address temperature (change) by lattice parameter (expansion), at the same time giving access to phonon modes [11,28,29] or sub-surface resolution.

We explore the limits of the method of Time-domain X-ray thermal scattering (TDXTS, name given in analogy to TDTR) to investigate the cross-plane thermal conductivity of layered systems. Two extreme structures serve as showcases, on the one hand isotopically modulated silicon multilayers [25,30] and on the other hand Mo/Si ML, respectively silicide(SiMo)/Si multilayer (ML) [31,32]. While the first have an overall high conductivity and low interface defect density, the latter have a high acoustic impedance mismatch and (due to sputter growth) more defects. It is demonstrated that the ’effective’ thermal conductivity of a layer stack can be determined in a wide range of values and separated from the unavoidable thermal interface resistance (TIR) at the interface between the sensing transducer layer and the investigated layer stack.

## 2. Materials and Methods

**Sample preparation:** The silicon-based multilayers on the basis of isotopically pure precursors (atomic mass Z${}_{Si}=$ 28, 29, 30 a. u.) were epitaxially grown by means of molecular beam epitaxy (MBE), respectively chemical vapor deposition (CVD, for the epilayer) on (100) oriented Si wafers of natural isotopic abundance (p-type substrates, boron doped, specific resistivity of 0.02 and 4 Ωcm, for the Z${}_{Si}=$ 29 and Z${}_{Si}=$ 30, respectively). The thickness of each individual isotope layer was set to 10 nm resulting in a total thickness of about 400 nm for the entire layer structure with 20 repeat structures. A reference structure of 400 nm silicon of natural abundance was grown as reference sample. In all cases the MBE, resp. CVD process started with the growth of an about 100 nm thick natural silicon buffer layer before the isotopic layer structure was deposited. Isotopic layering was verified by secondary ion mass spectroscopy (SIMS) [30], which gave a interface sharpness of <1 nm, defined by the precision in SIMS. The layers show a full epitaxy. X-ray truncation rod scattering at the (400) reflection (instrument SCD at KARA, KIT Karlsruhe, Germany) showed a modulation of intensity at the scattering vector shift corresponding to the periodicity, which implied a (9.7 + 9.7) nm layering with an isotopic strain of ±1.9 × 10${}^{-5}$, comparable to silicon isotopic lattice parameter differences [29]. The layer structure was verified by X-ray reflectivity at the synchrotron beamline SCD at KARA at an X-ray energy of 8.91 keV on a 6-circle diffractometer, using a linear pixel detector (Mythen, Dectris Ltd., Baden-Daettwil, Switzerland) as detection system.

Mo/Si and Mo${}_{0.85}$Si${}_{0.15}$/Si multilayers with 20 periods were deposited by magnetron sputter deposition. The experimental details for deposition of a single bilayer were reported in [32]. Mo/Si bilayer systems consist of an amorphous Si (a-Si) layer, and an amorphous or crystalline Mo layer. Under the present growth conditions, it was found that Mo crystallizes at a nominal thickness d(Mo) = 2.8 nm. Mo${}_{0.85}$Si${}_{0.15}$/Si shows a similar amorphous-crystalline transition at d(MoSi) = 4.0 nm. Due to the formation of an amorphous silicide interlayer with d ≃ 0.5–1 nm, in both cases the crystalline thickness is slightly lower than the nominal deposited layer thickness. For Mo/Si multilayers, silicide interlayers were found at both interfaces. The studied multilayers with sublayer ratio of 0.5 ± 0.05 were deposited on Si(100) wafers covered by native oxide, and capped with a Si layer (typical thickness 3 nm). The period of the multilayers was varied. The Mo deposition rate was 0.0307 nm/s, the Si deposition rate was 0.0125 nm/s. For the Mo/Si sample with 18.2 nm period, the Si rate was increased to 0.021 nm/s. The Mo${}_{0.85}$Si${}_{0.15}$/Si alloy layers were co-deposited from both targets. The Si deposition rate was reduced to 0.0042 nm/s, resulting in a total deposition rate of 0.0349 nm/s for Mo${}_{0.85}$Si${}_{0.15}$.

**Figure 1:**
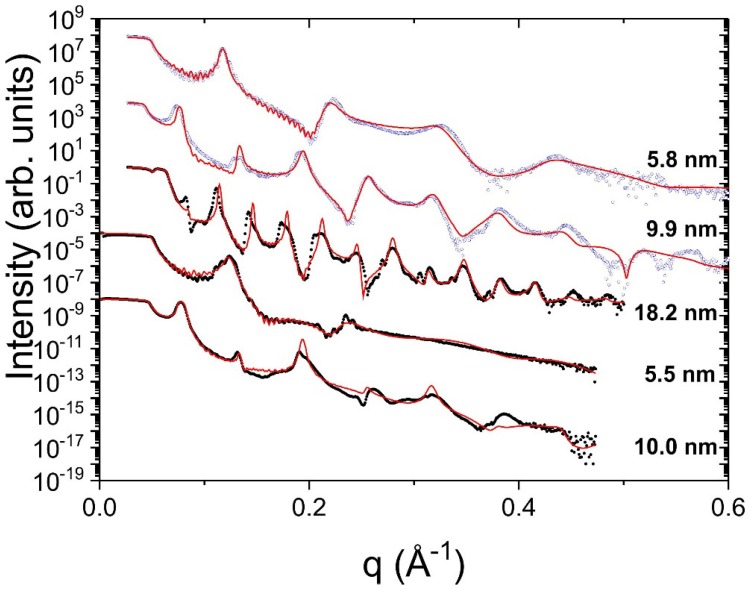
X-ray reflectivity measurements for the MoSi/Si (black dots) and Mo${}_{0.85}$Si${}_{0.15}$/Si (blue circles) multilayers. The period is indicated. The corresponding simulated XRR curves are shown as red lines.

Figure 1 shows XRR measurements of MoSi/Si (black dots) and Mo${}_{0.85}$Si${}_{0.15}$/Si (blue circles) multilayers with different period. For all samples, the higher order ML peaks are significantly broadened and dampened. This is mainly due to the ripple structure of the layers, which increases from period to period and is strongly correlated [33]. Comparing the Mo/Si and Mo${}_{0.85}$Si${}_{0.15}$/Si multilayers with 5.5 nm and 5.8 nm period, it is obvious that the multilayer peaks of Mo/Si are much more dampened. For bilayers, it was shown that below the crystallization threshold the roughness is comparable to the substrate roughness, but increases with layer thickness above the threshold. For all samples except of the 5.8 nm Mo${}_{0.85}$Si${}_{0.15}$/Si multilayer the Mo or Mo${}_{0.85}$Si${}_{0.15}$ layer thickness is above the crystallization threshold. The critical thickness d${}_{c}$ for crystallization is 2.8 nm for Mo and 4 nm for Mo${}_{0.85}$Si${}_{0.15}$ [32]. For the layers with period around 5.5 nm, the Mo layer is above d${}_{c}$, while the MoSi layer is below d${}_{c}$, explaining the larger damping of Mo. For 10 nm period, the difference is less pronounced since both layers are crystalline. The ML period and the Mo or MoSi fraction of the ML period were confirmed by fits of the measured XRR data. The fits were performed with the software GenX, using the Parratt formalism [34]. To simplify the stack model, it was assumed that the roughness increases linearly with the ML period. Each Mo or MoSi layer results in a stepwise roughness increase, but the silicon layers and the silicide interlayers only replicate the roughness of the layer below. Only for the Mo/Si ML with 5.5 nm period, the amorphous silicide interlayers at both interfaces were included in the fit. For all other samples, the interlayer signal was smeared out due to the layer corrugation and lower electron density contrast between Mo${}_{0.85}$Si${}_{0.15}$ and the silicide interlayer. For all samples, a surface roughness of the order of 1 nm was determined. The interlayer roughness increased up to 1–3 nm. These high values are caused by the ripple structure of the layers. However, a reliable quantification, based on the Parratt algorithm and a Gaussian roughness model is not possible any more since the roughness is correlated and comparable to the layer thickness.

**Time-domain X-ray thermal scattering:** one general approach of time-domain determination of thermal conductivity is to impulsively heat a transducer layer on top of the investigated layered structure and record its temporal cooling via an appropriate sensing probe. In established TDTR [20] a femtosecond laser is split between pump and probe pulses that can be delayed with respect to each other to probe the temporal temperature decay. An aluminum transducer usually serves as heater and temperature probe. Its optical reflectivity can be calibrated to derive the temperature. Aluminum shows a sufficiently linear thermal response for calibration. Laser focusing conditions (broad beams versus tight spots, or even thermal gratings) define whether the cross-plane conduction or also an in-plane conductivity component can be probed.

Pulsed X-ray scattering (and electron scattering) has shown to be a useful approach to determine thermal conductivity in layered structures [24,26,27,35,36]. In principle, TDXTS applies the same principles as TDTR above to determine the temperature of the top transducer via the thermal expansion coefficient of the material. While using synchrotron radiation for pulsed X-rays figures a rather involved approach, some advantages may concern the fact that thermal expansion can readily be quantified, elastic behavior can be distinguished from plastic deformation and the delay span of two independent sources can be extended from the picosecond to the millisecond range (as would also be possible with two independent lasers). Here we document the general approach and discuss the various aspects of real structures.

**Figure 2:**
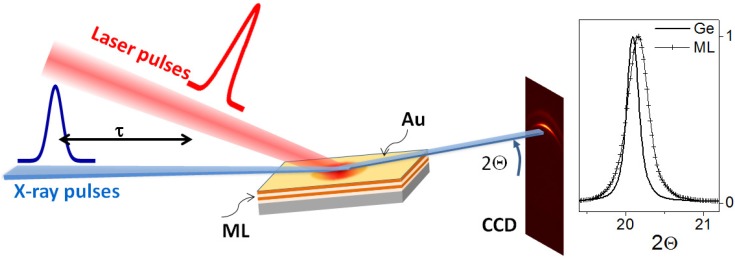
(**Left**) scheme of the laser pump, X-ray probe setup for the determination of cooling kinetics with 100 ps time resolution at a 3rd generation synchrotron. The train of X-ray and laser pulses are overlapped on the wafer surface with a defined delay τ between the pulses. Powder scattering is recorded on a CCD detector. Its angular deviation 2Θ(τ) serves as temperature monitor of the gold cover layer. (**Right**) Comparison of radial powder lines of the (111) reflection of gold in monochromatic X-ray mode (Ge monochromator) and in broad band mode (multilayer monochromator).

The pump-probe experiment was realized at the beamline ID09 at the European Synchrotron Radiation Facility (ESRF, Grenoble, France). A single-line undulator (U17) produces an X-ray beam that is pulsed with the filling mode of the electron bunches in the ring at a center energy of 15.2 keV. The X-rays are focused by a toroidal mirror onto the sample position to form a ≃0.1 mm sized focus, which elongates to 0.6 mm along the beam due to the shallow incidence. The frequency of the X-ray pulses arriving at the sample is reduced by a set of mechanical choppers (coarse heat load chopper and a microsecond high speed chopper at 986 Hz [37]). Further monochromatization is achieved by a Ge channel-cut crystal or a pair of multilayers (see discussion below). A femtosecond regenerative amplifier (Ti:Sa, Coherent Inc., Santa Clara, CA, USA) produces a laser pulse train synchronized to the X-ray pattern with a delay that can be modified in 5 ps steps up to 1 ms. The X-ray scattering is recorded on a (not time-resolving) CCD detector (MX170-HS, Rayonix LLC, Evanston, IL, USA) that accumulates the signal from typically 200 subsequent pulses at fixed delay after laser excitation. The acquisition is then repeated at a shifted delay to map the temporal evolution between delay τ=0 and <100 μs. The sketch in Figure 2 describes the approach. At a given laser fluence the position of laser-X-ray overlap is kept fixed on the sample surface.

Various metals can be employed as thermal transducer layers, as the thermal expansion from any suitable Bragg reflection of the layer can be recorded for determining the temperature. This can be done for single-crystalline transducers [24,38,39]. However, polycrystalline layers [25,36] simplify the alignment, as the incident Bragg angle has to be aligned only coarsely, while no scanning is required [40]. A typical image of the powder ring of gold is shown in Figure 2, as well as a radial profile as function of full scattering angle 2Θ (right side). We have investigated several materials to be used as transducers, such as silver, gold, platinum or aluminum. Gold has turned out to be the most suitable metal, as it has a high scattering cross section, is insensitive to atmospheric degradation and shows a strong crystalline texture. The latter represents the most important aspect as sputtered or evaporated gold thin films show preferential orientation of the lattice planes in <111> direction [41]. Evaporated films have displayed lateral peak widths of the (111) reflection of down to 2 degrees, while the sputtered films are typically more spread with a 8–10 degree orientation towards the surface normal. This orientation ensures a high scattering cross section with a selectivity towards cross-plane expansion. Silver is similarly suited, while displaying oxidation that may modify the properties and complicate modeling. The film thickness has to be balanced between thinnest films for achieving the highest time resolution and thus highest sensitivity on changes in the subsurface regions, in particular with overall high conductivity as in silicon, and a higher scattering cross section for thicker films. Additionally, thin films tend to de-wet at too high laser fluence. They also fail to fully localize the initial energy deposition in the metal transducer due to finite absorption cross section and injection of fast electrons into the sub-surface region [42,43]. We found an optimal gold thickness between 30 and 40 nm thickness. The layers used here were typically 44 nm thick.

Usually monochromatic X-rays would be employed in order to achieve the highest resolution in Bragg angle change due to thermal expansion, which just amounts for a few tenths of a degree at 2Θ of 20${}^{\circ }$. Nevertheless, the beamline ID09 offers using either a crystal monochromator (Ge channel cut in the present case) or a multilayer pair (consisting of Ru/B${}_{4}$C). The energy resolution of the monochromator ML together with the sharp undulator edge at 15.2 keV [44] allow for the preparation of an X-ray beam of 1.3% bandwidth [45]. While this results in a broadening of the typically >0.1${}^{\circ }$ reflections, the angular shift can still be resolved with the same or better precision due to higher counting statistics. Thus, a total of 2000 shots, or 2 s exposure time is sufficient for a temperature resolution below 0.5 K (see below). Data from the multilayers below is derived in both modes.

The near-infrared laser beam (800 nm, 2 ps) is focused to a size (0.5 mm) much larger than the X-ray footprint on the sample in order to achieve a homogeneous heating. Fluence is controlled by a motorized waveplate/polarizer combination to control absolute temperature rise. As discussed below, we restricted the fluence to a temperature rise of the gold film of below 150 K, typically only 80 K.

**Calculation of heat transfer:** The temporal cooling of the gold layer is related to energy loss due to heat conduction in contact to the underlying layered structure. Therefore radiation losses can be neglected. As such, the thermal conductivity of the structure can be deduced from the cooling speed. Ideally the analysis of the temporal behavior also allows for resolving depth-dependent properties. However, a proper modeling is required for a quantitative derivation. Here we assume a purely cross-plane heat flow, such that the systems are effectively modeled by 1-dimensional heat transfer. Importantly, any model has to incorporate the thermal interface resistance [19], as in particular the gold-semiconductor interfaces represent a strong obstacle against heat transfer. The so-called Kapitza resistance has been shown to be indispensable also for room temperature phenomena, as long as nanoscale processes are considered [23,46]. One approach is to solve the differential equations for diffusive heat transport by incorporating the effective heat flux at the boundaries of $\dot{Q}\propto A\cdot \Delta$T with the surface area A and the temperature drop ΔT across the interface [30]. Here, we follow an approach using Laplace space, that allows for a analytic formulation of conduction in layered systems for specific starting conditions, such as a uniformly heated transducer layer [47]. The so-called transmission-line approach can be extended for multiple interfaces [29,48,49].

(1) $$T\left(s\right)=\frac{Q\left(s\right)C_{11}}{e_{1}\sqrt{s}C_{21}},$$

with *T* being the temperature as function of the Laplace coordinate s, *Q(s)* being the Laplace form of the temporal heating source term (the laser profile) and *C* being the matrix product of (2 × 2) matrices containing the layers’ material parameters in the form $e_{i}=\sqrt{\rho_{i}c_{i}\kappa_{i}}$, the layer thickness $d_{i}$ and the TIR between each layer (with ρ density, *c* specific heat and κ thermal conductivity).

Individual layers are modeled by thermal conductance and diffusivity in a (2 × 2) matrix formalism. TIR can be introduced in a similar way. With this parametrization, the temperature in Laplace space is calculated by multiplying the matrices and deriving the temporal temperature decay of the transducer layer by backtransform. We use 3 or 4 layers, (1) gold top layer, (2–3) multilayer stack, and (4) substrate (semi-infinite). For details see [48,49].

Both the output of the Laplace approach and the solution of the diffusion equations scale with the absolute change in temperature through the source term. Explicit temperature dependence only enters via temperature-dependent material parameters, such as diffusivity or thermal expansion coefficient. Thus, a direct temperature dependence is not included in the Laplace approach.

In general, ρ, *c*, κ, d and the TIR could be free parameters in a fitting procedure. This would, however, be highly underdetermined, as for instance, the TIR and k are strongly interrelated parameters. In practice, $\kappa_{Si,nat}$ is taken from literature, the thicknesses of the layers are determined independently through X-ray reflectivity, respectively crystal truncation rod scattering and the densities are taken as bulk values. The TIR between gold and silicon for the isotope ML is determined from the reference sample. Therefore only one free parameter, the change of thermal conductivity of the ML stack relative to natural silicon remains to be optimized. The mean square error between the differences between reference sample and multilayer sample and the simulations of a native substrate and the effective conductivity of the simulated multilayer is taken as figure of merit. For the MoSi layers the TIR and the effective κ are fitted simultaneously, which is possible due to the strongly different time scales of cooling ruled by the TIR and the conductivity of the layer stack. As consequence, the determination of absolute values of the conductivity is less precise than the relative change between a reference sample and the investigated layer structure.

**Static characterization of thermal expansion coefficients:** The thermal expansion coefficient has been tested on a bulk sample, nanoparticles and the investigated films. The gold plate (99.95%, Chempur, Karlsruhe, Germany) as well as the gold nanoparticles were measured on a lab diffractometer with Cu anode(Rigaku Corp., Tokyo, Japan) equipped with a wafer heater (DHS1100, Anton Paar, Graz, Austria) and a linear detector (D/teX Ultra, Rigaku Corp.). The nanoparticles consisted of commercial spheres of 100 nm (BBInternational, Crumlin, UK) deposited in a single layer on a silicon wafer [50]. The organic coverage between substrate and particles prevents a lattice strain between particles and substrate. The gold film (44 nm) on top of the reference silicon wafer was analyzed at the beamline SCD, KARA by using a custom-made resistive heater with temperature control (331, Lake Shore Cryotronics, Inc., Westerville, OH, USA) and a linear detector (Mythen). In both cases, the temperature was ramped linearly at 1 K/min while recording the powder profile of the (111) reflection. The change in scattering angle is then converted to relative lattice expansion.

## 3. Results and Discussion

### 3.1. Thermal Expansion of Strained Thin Films

**Figure 3:**
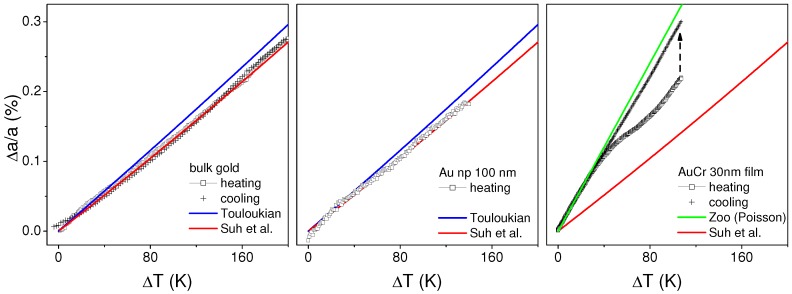
(**Left**) relative thermal expansion of a bulk gold plate as function of temperature rise above room temperature. The symbols mark heating ramp (dots) and cooling ramp (crosses). The lines are the calculated thermal expansion according to refs. [52] and [51]; (**Center**) relative thermal expansion of a single layer of spherical gold nanoparticles on a silicon wafer as function of temperature rise together with the calculated thermal expansion as in the left figure; (**Right**) relative thermal expansion of a sputtered gold film on silicon. The cooling curve is shifted for clarity to force overlap with the heating curve at ΔT = 0 K (indicated by the arrow). The lines are the calculated bulk thermal expansion from [52] as well as a calculation assuming additional Poisson expansion [54].

While the simulation of the thermal conductivity is inherently not temperature-dependent, the absolute temperature determination is nevertheless important for estimation of non-linear contributions of temperature on thermal strain. This could be a non-linear expansion coefficient, as well as plastic deformation. We have determined the lattice expansion coefficient of a bulk gold plate, gold nanoparticles and the used gold transducer layers. The thermal expansion coefficient of bulk gold is well known, while different parametrization yields different values. We have compared the lattice expansion of the gold plate in Figure 3 to two expressions given by Touloukian [51] and in ref. [52]. While the deviation from linearity is low, the power law parametrization to second order by Suh et al. fits our results better. In general, the expected expansion is well reproduced. We expect a similar behavior for finite-size gold particles, while a change of absolute expansion may take place for small particles due to modified interatomic potentials. This effect, however, is small if the particles size exceeds some tens of nanometers in gold [40]. The nanoparticles of 100 nm size and high crystallinity investigated here [53] show a thermal expansion coefficient that is well comparable to that of bulk gold (Figure 3, middle). This is reasonable, as due to the absorption process on the silicon surface the particles are coupled to the substrate only via organic ligand molecules, thus no epitaxial strain is supposed to occur. The cooling cycle in both cases is fully reversible.

The situation is different for a thin metal layer on a flat substrate. Although the growth via evaporation or sputtering does not result in an epitaxial relationship between the (oxidized) silicon substrate and the gold lattice, a strain is set to happen simply because the in-plane expansion needs to meet that of silicon, if no delamination is occurring. In fact, we used a thin chromium layer between silicon and gold for enhancing adhesion and lowering the TIR [41,55]. The out-of-plane expansion will be significantly modified, if the substrate possesses a significantly different expansion coefficient and the layer is clamped to it. The modified expansion coefficient can be rationalized as a compensation of in-plane restriction by Poisson deformation in the out-of-plane direction. Using the Poisson ratio ν the out-of-plane expansion $\alpha_{film}$ on a homogeneously heated layer system can be described as [54]:

(2) $$\alpha_{film}=\frac{\alpha_{gold}-\alpha_{Si}}{1-\nu }$$

Given a room-temperature thermal expansion of silicon (3 × 10${}^{-6}$/K), which is a factor of 5 smaller than that of gold (13.7 × 10${}^{-6}$/K in the present case), it is expected that the gold film will expand in excess in the out-of-plane direction. Indeed, we observe an initial coefficient of 3 × 10${}^{-5}$/K for the gold film, almost as high as the prediction in Equation (2). Earlier, a slight relaxation in in-plane direction was been observed [41], that may have been caused by the granular structure of the film.

The initial linear relation of lattice expansion with temperature is found to deviate towards a smaller slope at temperatures as low as 50 ${}^{\circ }C$. At higher temperature the initial slope is reached again. The subsequent cooling ramp, on the other hand, shows a good linear relationship. Such effects are seen prominently during the first temperature cycle, while they largely diminish in subsequent cycles (not shown). Therefore we attribute this behavior to plastic deformation, respectively annealing of defects during growth. It is a common observation that polycrystalline films are under in-plane compressive strain. This strain can relax upon annealing to reduce the out-of-plane expansion [56]. Practically, such a non-linear relation would be a strong hindrance for using such layers as temperature sensors (including possibly TDTR). In following we will show that the effect is under control, when (i) limiting the temperature rise and (ii) correcting for the plastic deformation, which gradually builds up with laser exposure. The plastic deformation is quantified during the time-resolved measurements with interleaved measurements at negative delay.

For time-resolved heating, a similar uni-axial expansion of the gold film is expected, with an even higher out-of-plane coefficient, given that the silicon substrate heats up even less due to fast heat dissipation.

For photo-excitation conditions of a gold film Nicoul et al. [36] have given a relationship between temperature rise based on the model by Thomsen [57], which suggests an even higher increase of the out-of-plane expansion coefficient, which we, however, can not confirm.

### 3.2. Heat Transfer in Epitaxial Isotopically Modulated Multilayers

**Figure 4:**
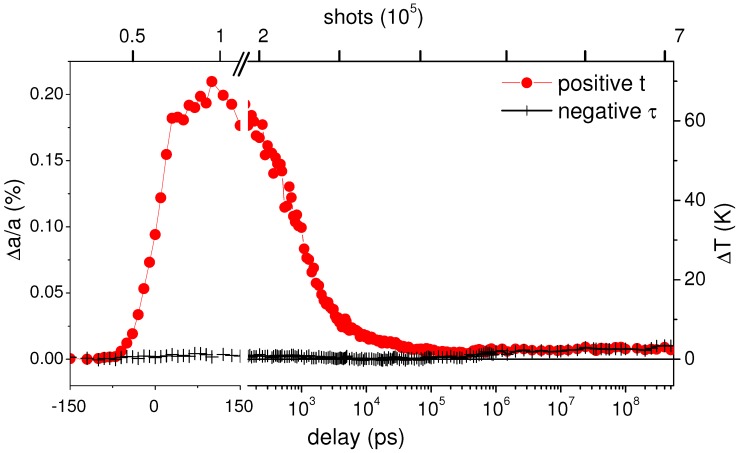
Lattice thermal expansion of a gold film on top of a natural silicon wafer as function of delay between laser and X-ray probe pulse. The circles mark an acquisition, where the delay has been constantly increased after each measuring point, while the delay has been kept constant at −2 ns for the data with crosses, but directly preceding the acquisition with varied delay. The corresponding number of laser shots accumulated on the illuminated spot is marked on the top axis.

**Figure 5:**
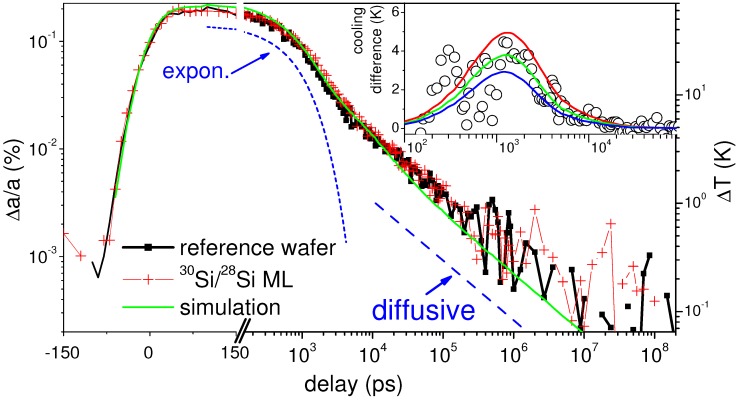
Lattice expansion of the gold layer on top of two silicon samples, the reference substrate (black squares) and the wafer with the isotope-modulated multilayer stack (red crosses). The lines are a guide to exponential and diffusive decay, respectively simulations of temperature decay based on the layer model in conductivity. The shape around τ= 0 is modeled by a sigmoidal function with 30 ps Gaussian width. The inset displays the change in cooling of the ${}^{30}$Si/${}^{28}$Si-ML compared to the native Si reference sample. The lines are simulations by assuming a change of thermal conductivity to 55 (red), 60 (green) and 65 (blue) W/(m·K), from top.

A typical decay of the thermal lattice expansion following photo-excitation is displayed in Figure 4. For negative delays, the probe pulse precedes the laser pump pulse so that no expansion is observed. This changes while the delay enters the pulse length of the X-ray pulse, where a gradual increase of expansion is seen that saturates at a 100 ps delay. Thereafter, the expansion decays with a particular characteristics. The first 1–2 ns delay span is described by an exponential decay with a lifetime around 1.2 ns (see Figure 5), followed by a slowing down of relaxation (and thus cooling). The final decay after some 100 nanoseconds approaches a square-root form. The exponential decay is explained by the predominance of the TIR in a situation, where the gold film is warm, while the silicon substrate is still cold. In the TIR case the absolute flux is proportional to the difference in temperature, thus an integration yields an exponential decay. The square-root behavior is clearly linked to bulk diffusion, as found in Fick’s law. On the nanosecond time scale the two cooling regimes are balanced.

At the same time, a measurement of expansion at negative decay before each positive delay point shows a variation in absolute value, which would not be expected for a fully reversible situation. The delay axis for these reference measurements is in fact an exposure scale (as indicated on the top axis). With ongoing exposure the expansion first turns negative, while at a later stage is becomes positive again. Keep in mind that the each data point encompasses 5000 individual laser pulses, but at fixed fluence, so that the end temperature is reached thousands of times with ongoing exposure. In other samples the detailed course of the irreversible changes would differ in its exact shape. Therefore we interpret this exposure behavior as a plastic deformation in a similar fashion as observed in the steady-state heating above. This is rationalized by the notion that expansion at positive delay follows that at negative delay for the last measurement points, where the reversible heating of the gold film has decayed. In both cases the plastic deformation remains. An effect by a slow warming of the sample by the series of laser pulses is also possible, but accounts only for several Kelvin at 1 kHz repetition rate [58]. Additionally, the equilibrium will be reached within the first hundreds of laser shots, so that a drift in the expansion baseline (−2 ns) would occur within the first few data points. In order to derive the true time-resolved expansion the contribution due to plastic deformation is subtracted.

This correction yields a precise decay of lattice heating shown in Figure 5 where the late-time square-root decay can be reproduced in good agreement to the calculations. The axis on the right indicates the absolute temperature change according to Equation (2). Figure 5 displays both the cooling of the native silicon reference sample as well as that of a 20 × (10 + 10) nm isotope-modulated stack of ${}^{28}$Si and ${}^{30}$Si. In both cases the initial exponential as well as the final square-root decay coincide. This is rationalized by a comparable TIR value of both samples as well as similar bulk heat diffusion into the (same) substrate. As consequence, it is possible to fix the TIR value in both cases to extract the change in effective thermal conductivity of the stack as single parameter. Cooling is delayed slightly for the multilayered sample, which qualitatively points towards a lower conductivity. Nevertheless, a quantitative determination relies on the modeling of heat transfer. The best value for the multilayer conductivity is (61 ± 10) W/(m·K) as compared to that of the reference sample of (130 ± 25) W/(m·K). Earlier, we have reported slightly different values in the present system (compare also Table 1). We attribute this to the different gold film thickness. Here, we have chosen 44 nm thick layers as compared to the <30 nm [43] or 80 nm [25] earlier. This avoids on the one hand leaking of fast electrons (or laser light) into the silicon stack and on the other hand sensitivity to absolute high values of thermal conductivity is preserved by keeping the gold layer as thin as possible.

An in-depth discussion of the interpretation of thermal conductivity in complex tailored systems is beyond the scope of the present work. In general, heat can be transported by the conduction electrons as well as phonons. In our low-doped silicon samples electron conduction at room temperature plays a minor role. Limitations to heat conduction by phonons are given by several effects that limit the mean-free path of phonons and thus represent an obstacle to transport. The conventional mechanisms described are incoherent ones, where a phonon is scattered at point defects or an interface or by multiphonon process (Umklapp processes). In well-ordered multilayers, on the other hand coherent phonon interaction across the interfaces can also take place. These are described to cause partial phonon band blockage or even Bloch-like pass bands. Recent lattice dynamics also point towards mini-Umklapp processes in periodic multilayers [29].

Simulations of the cross-over between coherent and incoherent transport in the present silicon isotope multilayers shall illustrate the expected behavior. Frieling et al. have conducted non-equilibrium molecular dynamics simulations (NE-MD) of heat transfer across a stack of mass-modulated silicon layers [59,60]. NE-MD calculations of the thermal conductivity of natural Si were also performed for comparison. Details on the calculations and on the MD simulation cell are given in Ref. [59].

**Figure 6:**
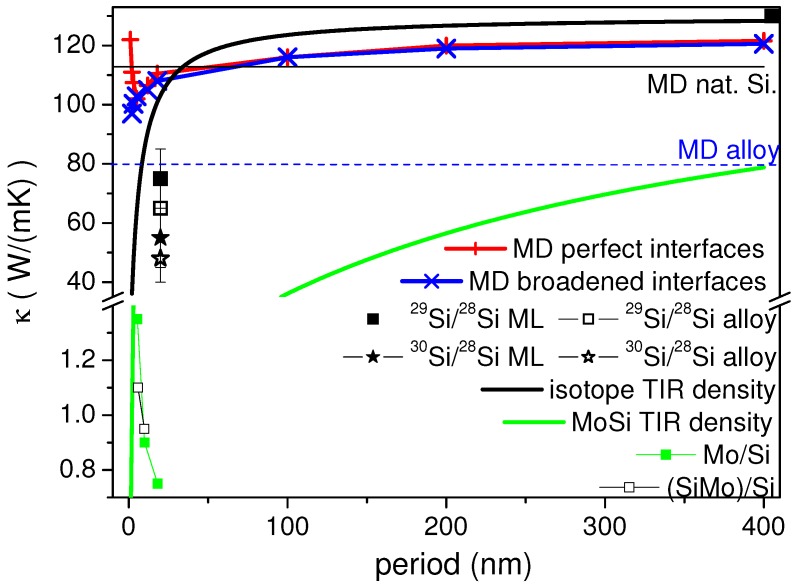
Comparison of the measured thermal conductivities of the silicon isotope multilayers of molecular dynamics calculations as a function of the periodicity of the stack (Frieling et al., [59]). The horizontal lines mark the simulation result for natural silicon (black line) and for the random ${}^{28}$Si${}_{0.5}{/}^{30}$Si${}_{0.5}$ alloy (blue dashed line). The lower part of the figure contains experimental data from the Si/Mo multilayers. The curved lines indicate an effective conductivity as a sum of TIR of the individual layers for the isotope modulation and the Si/Mo stacks (lower green thick lines), respectively.

Figure 6 reproduces the main results. The effective thermal conductivity starts by dropping from the bulk value of 112 W/(m·K) at very large period, thus only a few interfaces are sensed by the phonons. The simulated bulk conductivity is lower than the experimental one due to the finite size of the simulation box [60]. When reducing the period, the conductivity drops, essentially because the interface density increases. The product of TIR at each interface and the layer thickness qualitatively reproduces this monotonously decaying function with reduced period. The TIR value has been taken from [30]. However, below a period of 6 nm the conductivity rises again for the ideal ML with perfect interfaces. On this length scale the phonon band structure for long-wavelength phonons does not sense the periodicity any more, thus marking a transition from incoherent to coherent transport.

On the other hand, point defects scatter phonons effectively as well. This can already be seen for the structure with gradual mixing between the layers (for details see [59]). When rearranging all the isotopes in the layers into arbitrary positions (which has been done experimentally by annealing the ML at 950 ${}^{\circ }C$ for 120 h) one obtains a homogeneous alloy, however with disordered isotope distribution. In this situation the simulated thermal conductivity drops further.

Comparing the prediction to our data we find that the drop in conductivity from the natural silicon is higher than in the MD, but follows the same order of magnitude, the drop for the ${}^{30}$Si/${}^{28}$Si being higher than that of the ${}^{29}$Si/${}^{28}$Si ML. Forming an alloy from these layers additionally reduces conductivity further (see also Table 1). Interestingly this further drop is less pronounced in the experiment than predicted by the MD simulations. This points towards defects already playing a role for the MBE-grown layers as compared to the ideal situation in MD.

The precision of the evaluation of conductivity is naturally limited by the resolution in temperature rise and thus by counting statistics of the scattering yield (see inset to Figure 5). Additionally, the high absolute conductivity of the system poses a particular problem to the time-domain approach. As the quantification of conductivity is based on determining the cooling rate, it is limited by other sources of thermal resistance in the system. The first and most important one is the TIR at the interface gold-layer, which limits heat transfer. With a high TIR, the changes in cooling at later times than the exponential decay in Figure 5 become marginal. A similar influence may be imposed by the conductivity of the substrate. On a substrate with very low conductivity the heat may be kept in the layer system, modifying the residence time. In the current approach the cooling was followed over several decades in delay, such that resistance may be localized in depth to a certain extent. Nevertheless, determination of conductivities above 100–150 W/(m·K) is restricted with the present setup, unless a lower TIR at the gold-layer interface can be achieved. At the same time, lower TIR often correlates with lower Schottky barrier, such that non-thermal electrons might escape into the layer system. In that case, the thermal conduction would have to be modeled in a more detailed way.

A critical assessment of absolute and relative errors of the evaluation of κ reveals that relative changes of analogous samples as listed in Table 1 can be rather small, while the absolute determination of the conductivity of the reference sample with natural silicon is strongly related to other optimization parameters, such as the TIR. Earlier, a direct solution of the differential equations of thermal transport has been used [43], which allowed to incorporate direct energy deposition into the layer by a leaky gold film or fast electrons. There, the TIR was found to be lower, while the conductivity of native silicon was higher. Still, the relative reduction of conductivity in the multilayers was similar.

### 3.3. Heat Transfer in Sputtered Molybdenum Silicon Multilayers

Mo/Si multilayers represent the opposite case as compared to the isotope layers, which show low density contrast across the interfaces. The density difference between molybdenum and silicon results in a much lower effective thermal conductivity of the layer system. Additionally, interlayers of amorphous silicon, respectively silicide may reduce thermal conductivity further. Bozorg-Grayeli et al. [31] investigated Si-Mo multilayer structures to find a thermal conductivity as low as 1.1 W/(m·K) and additionally observed the increase of conductivity of a molybdenum silicide film with annealing from 2 to 3 W/(m·K) as following the irreversible amorphous-crystalline transition. Furthermore the conductivity of such multilayers has been investigated in terms of cross-coupling between electronic and phononic sub-systems [61], which opens a further pathways for heat conduction. A detailed model predicted 1.3–1.5 W/(m·K), which matches the observed 1.2–1.4 W/(m·K).

**Figure 7:**
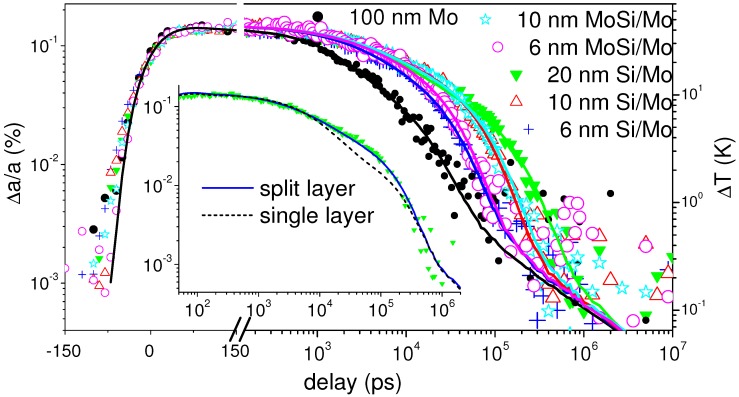
Lattice expansion of the gold layer on top of Si-Mo multilayer samples, as well as a 100 nm molybdenum layer on silicon substrate (black circles). The lines are results of a best fit with the heat transfer model with an effective thermal conductivity modeled independently for two splitted parts of the stack. The inset compares the two-layer fit with a single-layer fit.

The TDXTS data (Figure 7) on the set of multilayer structures investigated here shows that cooling in general is much slower for the MoSi system than for the isotope samples with the limit of bulk conductivity (as seen by the change from the power-law to the diffusive limit) not being reached before 1 μs. The thermal decay of the multilayers is found to be an order of magnitude slower than the decay of the molybdenum layer. Results of the calculations are found in Table 1. The 100 nm molybdenum layer shows a conductivity 80 W/(m·K), again limited in precision by the large TIR, comparable to tabulated values. In contrast, the conductivity of the ML is lowered to 0.75 to 1.35 W/(m·K). A fit with a single ML layer with effective conductivity is only reproducing the time scale, while some deviations are still seen Figure 7. A better fit can be achieved by dividing the ML thickness in two equal regions and allowing the conductivity to vary independently. In that case the model fit is much better, but in all cases suggests that the lower part of the ML has a higher conductivity (of 2–4 W/(m·K)) than the top part. This points towards the increase in disorder and ripple effect disturbing the layering and reducing effective conductivity.

Overall, it is found that the thermal conductivity is lowest for the larger periods as well as for the silicide ML. A generic diffuse mismatch mode with counting interfaces would predict the opposite. The comparison with an effective conductivity for a typical TIR of (heavy) metal-silicon interfaces [41,62] of 10${}^{-9}$ m${}^{2}\cdot$K/W in Figure 6 shows that the effective conductivity is in the same range, while the thickness dependence is not reproduced. However, a periodicity of 6, respectively 10 nm, can already be too short for having an effect on long-wavelength phonons, as has been predicted by theoretical considerations [15,63] and simulations [59,64]. More likely, in view of the lower conductivity of the silicide ML, the real structure plays a central role in the magnitude of suppression of heat conduction. For the lowest periods the intermixing at the interface and the lateral variation in periodicity can lead to an effectively higher conductivity. The silicide ML in general show a better definition of density variation. Despite the lower nominal TIR at each (SiMo)-Si interface the effective conductivity is lower.

## 4. Conclusions and Outlook

It has been demonstrated that time-resolved X-ray powder scattering of thermal transducer layers on top of a layered surface can be used to resolve and quantify the cross-plane thermal conductivity of the materials. It is possible to discern between TIR at the transducer interface and contributions from thermal conductivity. Synchrotron-based diffraction with sub-nanosecond resolution is particularly suited to follow thermal kinetics over several decades in time and temperature, thus allowing address as well the depth of where a resistance in heat flow occurs. While cooling is a diffusive phenomenon a limited depth resolution may still be achieved.

The methodology is analogous to the established TDTR, using a purely optical pump-probe approach. Meanwhile, in X-ray scattering understanding the atomic scale structure, plastic deformation processes and obtaining a absolute temperature calibration are straightforward. The delay range spans 100 ps up to millisecond times, which allows visualizing different heat conduction regimes. The transducer film has to be optimized in order to minimize the TIR at the interface to the probed thin films as well as in thickness. Thinnest films below 30 nm do not dissipate the total laser energy in the film, while thicker films lengthen the time scale for heat transfer into the thin films and thus reduce sensitivity for high conductivity in the probed structures. Gold has been found to be ideal due to its strong preferential crystal orientation for various growth methods, such as thermal evaporation or sputtering.

Nevertheless, the described method relies on access to highly specialized synchrotron beamlines. It would possibly not be used for routine characterization, but rather for selected advanced problems. A simpler approach may meanwhile be accessible on any synchrotron beamline, taking advantage of developments in detector technology [65] and data processing. With avalanche photo-detectors a time resolution on the nanosecond scale can be achieved [66,67]. This would allow recording shifts in powder peak position of the gold layer without a dedicated pump-probe beamline. In a pump-record approach the laser pump pulse represents the start signal for a time-resolved acquisition of scattering intensity while the x-ray emitter (also preferably a synchrotron) will serve as continuous source. A time-resolved linear detector will allow for a resolution of the powder profile, while a point detector with knife-edge discrimination may already suffice to quantify the amount of peak shift and thus lattice expansion. At the same time, the pump-record approach would minimize the influence of plastic deformation in a similar way as the lock-in approach of TDTR. Even laboratory sources based on plasma-generated X-rays or liquid anodes may deliver sufficient pulsed flux for meaningful data collection [10,68].

## Figures and Tables

**Table 1:** List of all used thin-film structures with the multilayer period, TIR at the interface gold-layer and the determined effective thermal conductivity of the full layer stack.

Sample	Period	TIR (m·K/W)	$\kappa_{1}$ (W/(m·K))
nat-Si	–	4.5 × 10${}^{-9}$	130
28-Si/29-Si	20 nm	4.5 × 10${}^{-9}$	(81 ± 10)
28-Si/29-Si	alloy	4.5 × 10${}^{-9}$	(79 ± 10)
28-Si/30-Si	19.4 nm	4.5 × 10${}^{-9}$	(61 ± 10)
28-Si/30-Si	alloy	4.5 × 10${}^{-9}$	(51 ± 8)
100 nm Mo	–	2.5 × 10${}^{-8}$	(80 ± 20)
Mo/Si	5.5 nm	2 × 10${}^{-8}$	(1.35 ± 0.2)
Mo/Si	10 nm	2 × 10${}^{-8}$	(0.9 ± 0.15)
Mo/Si	18.2 nm	2.2 × 10${}^{-8}$	(0.75 ± 0.15)
(SiMo)/Si	5.8 nm	2 × 10${}^{-8}$	(1.1 ± 0.2)
(SiMo)/Si	9.9 nm	2 × 10${}^{-8}$	(0.95 ± 0.2)

## Abbreviations

The following abbreviations are used in this manuscript:

TDTR	Time-Domain Thermal Reflection
TDXTS	Time-domain X-ray Thermal Scattering
XRD	X-ray Diffraction
XRR	X-ray Reflectivity
ML	Multilayer
d${}_{c}$	critical thickness for crystallization
TIR	thermal interface resistance
κ	(effective) thermal conductivity
α	linear thermal expansion coefficient
ν	Poisson ratio
CVD	chemical vapor deposition
MBE	molecular beam epitaxy
NE-MD	non-equilibrium molecular dynamics simulations
